# Supplementary material for: Genome-wide identification, characterization and gene expression of BES1 transcription factor family in grapevine (Vitis vinifera L.)
Source: Sci Rep. 2023 Jan 5;13:240. doi: 10.1038/s41598-022-24407-y (PMC9816167; doi:10.1038/s41598-022-24407-y)
Supplement: Supplementary file 3 — Supplementary Information. [file 41598_2022_24407_MOESM3_ESM.zip › Vvi_Atr/Vitis_vinifera.PN40024.v4.dna_sm.toplevel.fa.vs.Amborella_trichopoda.AMTR1.0.dna_sm.toplevel.fa.html/Atr-AmTr_v1.0_scaffold00175.html]

|  |  |  |  |  |  |  |  |  |  |  |  |  |  |
| --- | --- | --- | --- | --- | --- | --- | --- | --- | --- | --- | --- | --- | --- |
| Duplication depth | Reference chromosome | Collinear blocks | | | | | | | | | | | |
| 0 | Atr-ERN15946 |  |  |  |  |  |  |  |  |  |  |  |  |
| 0 | Atr-ERN15947 |  |  |  |  |  |  |  |  |  |  |  |  |
| 0 | Atr-ERN15948 |  |  |  |  |  |  |  |  |  |  |  |  |
| 0 | Atr-ERN15949 |  |  |  |  |  |  |  |  |  |  |  |  |
| 0 | Atr-ERN15950 |  |  |  |  |  |  |  |  |  |  |  |  |
| 0 | Atr-ERN15951 |  |  |  |  |  |  |  |  |  |  |  |  |
| 0 | Atr-ERN15952 |  |  |  |  |  |  |  |  |  |  |  |  |
| 1 | Atr-ERN15953 |  | Vvi-Vitvi06g00300\_t001 |  |  |  |  |  |  |  |  |  |  |
| 1 | Atr-ERN15954 |  | Vvi-Vitvi06g00299\_t001 |  |  |  |  |  |  |  |  |  |  |
| 2 | Atr-ERN15955 |  | \| \| |  | Vvi-Vitvi13g00588\_t001 |  |  |  |  |  |  |  |  |
| 2 | Atr-ERN15956 |  | \| \| |  | Vvi-Vitvi13g00589\_t001 |  |  |  |  |  |  |  |  |
| 2 | Atr-ERN15957 |  | \| \| |  | \| \| |  |  |  |  |  |  |  |  |
| 2 | Atr-ERN15958 |  | Vvi-Vitvi06g00298\_t001 |  | \| \| |  |  |  |  |  |  |  |  |
| 2 | Atr-ERN15959 |  | \| \| |  | Vvi-Vitvi13g00590\_t001 |  |  |  |  |  |  |  |  |
| 2 | Atr-ERN15960 |  | \| \| |  | \| \| |  |  |  |  |  |  |  |  |
| 2 | Atr-ERN15961 |  | Vvi-Vitvi06g00296\_t002 |  | \| \| |  |  |  |  |  |  |  |  |
| 2 | Atr-ERN15962 |  | Vvi-Vitvi06g00295\_t001 |  | \| \| |  |  |  |  |  |  |  |  |
| 2 | Atr-ERN15963 |  | \| \| |  | Vvi-Vitvi13g00592\_t001 |  |  |  |  |  |  |  |  |
| 2 | Atr-ERN15964 |  | Vvi-Vitvi06g00294\_t001 |  | \| \| |  |  |  |  |  |  |  |  |
| 2 | Atr-ERN15965 |  | Vvi-Vitvi06g00293\_t001 |  | Vvi-Vitvi13g00597\_t002 |  |  |  |  |  |  |  |  |
| 2 | Atr-ERN15966 |  | Vvi-Vitvi06g00292\_t001 |  | \| \| |  |  |  |  |  |  |  |  |
| 2 | Atr-ERN15967 |  | \| \| |  | Vvi-Vitvi13g00598\_t001 |  |  |  |  |  |  |  |  |
| 2 | Atr-ERN15968 |  | Vvi-Vitvi06g00290\_t001 |  | \| \| |  |  |  |  |  |  |  |  |
| 2 | Atr-ERN15969 |  | Vvi-Vitvi06g00289\_t001 |  | \| \| |  |  |  |  |  |  |  |  |
| 2 | Atr-ERN15970 |  | Vvi-Vitvi06g00288\_t001 |  | \| \| |  |  |  |  |  |  |  |  |
| 2 | Atr-ERN15971 |  | \| \| |  | \| \| |  |  |  |  |  |  |  |  |
| 2 | Atr-ERN15972 |  | Vvi-Vitvi06g00287\_t001 |  | \| \| |  |  |  |  |  |  |  |  |
| 2 | Atr-ERN15973 |  | \| \| |  | \| \| |  |  |  |  |  |  |  |  |
| 2 | Atr-ERN15974 |  | \| \| |  | Vvi-Vitvi13g00600\_t001 |  |  |  |  |  |  |  |  |
| 2 | Atr-ERN15975 |  | Vvi-Vitvi06g00286\_t001 |  | \| \| |  |  |  |  |  |  |  |  |
| 2 | Atr-ERN15976 |  | \| \| |  | Vvi-Vitvi13g00601\_t001 |  |  |  |  |  |  |  |  |
| 2 | Atr-ERN15977 |  | \| \| |  | \| \| |  |  |  |  |  |  |  |  |
| 2 | Atr-ERN15978 |  | \| \| |  | \| \| |  |  |  |  |  |  |  |  |
| 2 | Atr-ERN15979 |  | \| \| |  | \| \| |  |  |  |  |  |  |  |  |
| 2 | Atr-ERN15980 |  | Vvi-Vitvi06g00285\_t001 |  | \| \| |  |  |  |  |  |  |  |  |
| 2 | Atr-ERN15981 |  | \| \| |  | \| \| |  |  |  |  |  |  |  |  |
| 2 | Atr-ERN15982 |  | Vvi-Vitvi06g00284\_t001 |  | Vvi-Vitvi13g00602\_t001 |  |  |  |  |  |  |  |  |
| 1 | Atr-ERN15983 |  |  |  | Vvi-Vitvi13g00604\_t001 |  |  |  |  |  |  |  |  |
